# The MASP Family of *Trypanosoma cruzi*: Changes in Gene Expression and Antigenic Profile during the Acute Phase of Experimental Infection

**DOI:** 10.1371/journal.pntd.0001779

**Published:** 2012-08-14

**Authors:** Sara Lopes dos Santos, Leandro Martins Freitas, Francisco Pereira Lobo, Gabriela Flávia Rodrigues-Luiz, Tiago Antônio de Oliveira Mendes, Anny Carolline Silva Oliveira, Luciana Oliveira Andrade, Égler Chiari, Ricardo Tostes Gazzinelli, Santuza Maria Ribeiro Teixeira, Ricardo Toshio Fujiwara, Daniella Castanheira Bartholomeu

**Affiliations:** 1 Departamento de Parasitologia, Universidade Federal de Minas Gerais, Minas Gerais, Brazil; 2 Departamento de Morfologia, Universidade Federal de Minas Gerais, Minas Gerais, Brazil; 3 Departamento de Bioquímica e Imunologia, Universidade Federal de Minas Gerais, Minas Gerais, Brazil; Universidad Autónoma de Yucatán, Mexico

## Abstract

**Background:**

*Trypanosoma cruzi* is the etiological agent of Chagas disease, a debilitating illness that affects millions of people in the Americas. A major finding of the *T. cruzi* genome project was the discovery of a novel multigene family composed of approximately 1,300 genes that encode mucin-associated surface proteins (MASPs). The high level of polymorphism of the MASP family associated with its localization at the surface of infective forms of the parasite suggests that MASP participates in host–parasite interactions. We speculate that the large repertoire of MASP sequences may contribute to the ability of *T. cruzi* to infect several host cell types and/or participate in host immune evasion mechanisms.

**Methods:**

By sequencing seven cDNA libraries, we analyzed the MASP expression profile in trypomastigotes derived from distinct host cells and after sequential passages in acutely infected mice. Additionally, to investigate the MASP antigenic profile, we performed B-cell epitope prediction on MASP proteins and designed a MASP-specific peptide array with 110 putative epitopes, which was screened with sera from acutely infected mice.

**Findings and Conclusions:**

We observed differential expression of a few MASP genes between trypomastigotes derived from epithelial and myoblast cell lines. The more pronounced MASP expression changes were observed between bloodstream and tissue-culture trypomastigotes and between bloodstream forms from sequential passages in acutely infected mice. Moreover, we demonstrated that different MASP members were expressed during the acute *T. cruzi* infection and constitute parasite antigens that are recognized by IgG and IgM antibodies. We also found that distinct MASP peptides could trigger different antibody responses and that the antibody level against a given peptide may vary after sequential passages in mice. We speculate that changes in the large repertoire of MASP antigenic peptides during an infection may contribute to the evasion of host immune responses during the acute phase of Chagas disease.

## Author Summary

The parasite *Trypanosoma cruzi* is the etiologic agent of Chagas disease, a neglected tropical disease. A major finding of the *T. cruzi* genome project was the discovery of a multigene family that encodes mucin-associated surface proteins (MASP), a highly polymorphic family expressed at the surface of infective forms of the parasite. We speculate that MASP may contribute to the ability of *T. cruzi* to infect several host cells and/or participate in host immune evasion mechanisms. To begin investigating this hypothesis, we analyzed the MASP expression profile in trypomastigotes derived from different host cells and in bloodstream parasites after sequential passages in mice. We also investigated the MASP antigenic profile in acutely infected mice. We observed more pronounced MASP expression changes by comparing bloodstream and tissueculture trypomastigotes and between bloodstream forms from sequential passages in infected mice. We also found that MASP peptides could trigger different IgG and IgM antibody responses and that the antibody level against a given peptide may vary after sequential passages in mice. We speculate that changes in the large repertoire of MASP antigenic peptides during the course of an infection may contribute to the evasion of host immune responses during the acute phase of Chagas disease.

## Introduction

*Trypanosoma cruzi* is the etiological agent of Chagas disease, a major public health problem in Central and South America. Currently there are approximately 10 million people infected and 40 million people at risk of acquiring the disease [1], [2]. Trypomastigotes are the bloodstream circulating form that infect a wide variety of nucleated host cells and subsequently differentiate into the intracellular replicative amastigote forms. After several rounds of binary division, amastigotes differentiate into trypomastigotes, which are released into the extracellular medium and bloodstream. The repetitive cycle of cell infection triggers the acute phase of Chagas disease, characterized by high blood parasitaemia, broad tissue parasitism, and a strong host immune response. The chronic phase is achieved after the host immune system controls the parasitaemia but fails to completely eliminate the parasite [3].

The annotation of the *T. cruzi* genome revealed a new multigene family composed of approximately 1,300 genes, which became known as mucin-associated surface protein (MASP) as they were not randomly distributed throughout the genome but instead clustered with genes encoding mucins and other surface protein families [4]. A previous study on the molecular characterization of a few members found that MASP proteins are expressed on the surface of the circulating infective forms of the parasite and can be shed into the extracellular medium [5]. MASP expression in the trypomastigote stage was also demonstrated by recent proteomic studies [6], [7]. Moreover, the MASP family is characterized by a strikingly variable and repetitive central region composed of peptides shared among its members, thus contributing to the extended repertoire of parasite polypeptides that could be exposed to the host cells and immune system [5]. The MASP repertoire of peptides could also contribute to cell-type-specific interactions, because the polymorphism of *T. cruzi* surface proteins has been suggested to be an important factor in the parasite's ability to infect multiple cell types [8], [9]. Taken together, this evidence has prompted us to examine a possible role of the MASP family in host–parasite interactions, such as the host-cell-dependent expression profile or/and immune evasion mechanisms. We observed differential expression of a few MASP genes between trypomastigotes derived from epithelial and myoblast cell lines. The more pronounced MASP expression changes were observed when comparing bloodstream and tissue-culture trypomastigotes and between bloodstream forms from sequential passages in acutely infected mice. Furthermore, in this work we describe the antibody recognition of several MASP peptides. Taken together, our findings prompt us to speculate that variations in the large repertoire of antigenic peptides derived from the MASP family may favor the parasite's escaping the host immune response during the acute phase of infection.

## Methods

### Trypomastigote culture, collection, and RNA extraction

**Figure 1:**
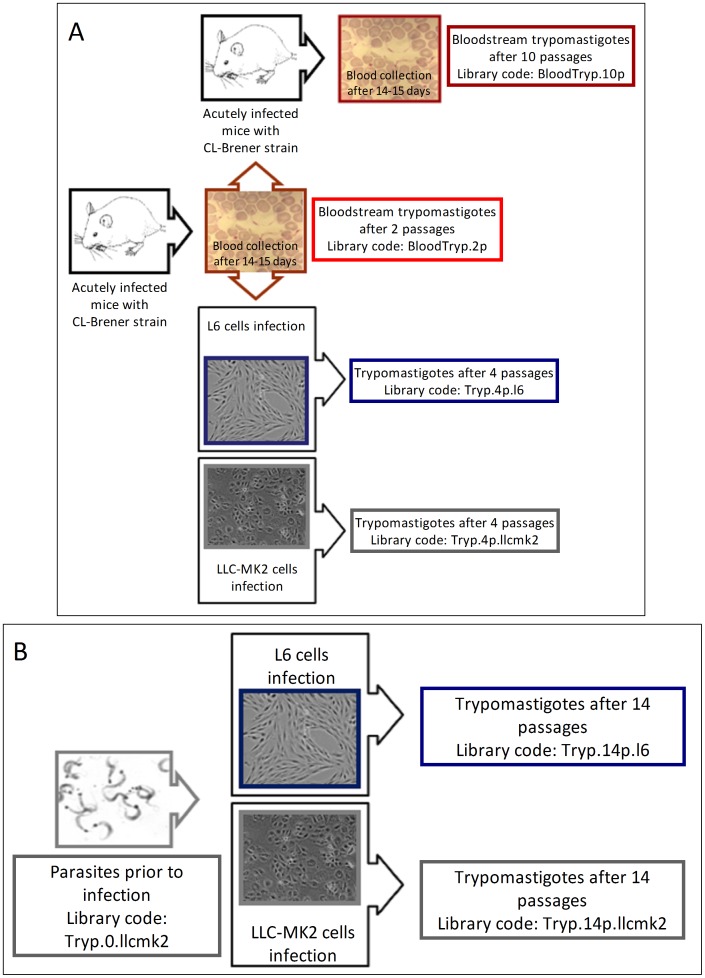
Experimental models. **A**: Mouse groups were sequentially infected with CL Brener clone. Blood collections were performed after 14–15 days. Bloodstream parasite forms were collected after two and ten passages. Part of the trypomastigotes isolated from mouse blood was used in LLC-MK2 and L6 cell infection (bloodstream parasite forms after two passages in mice). Trypomastigotes were collected after four sequential passages in culture. **B**: The same group of culture trypomastigotes was used in the infection of LLC-MK2 (epithelial) and L6 (myoblast) cell types. Trypomastigotes were collected after 14 sequential passages in culture.

The following steps for CL Brener trypomastigote culture and collection are detailed in Figure 1.

#### *In vitro* culture of trypomastigotes

CL Brener clone trypomastigotes obtained from one culture flask were used to infect two cell lineages, the rhesus-monkey epithelial cells LLC-MK2 and the rat myoblasts L6, both grown at 37°C, 5% CO_2_ in RPMI medium supplemented with 1% fetal bovine serum.

#### *In vivo* production of trypomastigotes

To obtain bloodstream trypomastigotes, 10 Swiss mice were infected intraperitoneally with 50,000 CL Brener trypomastigotes, and then bled after 14–15 days when they reached the peak of parasitaemia. Bloodstream parasite forms were collected after two and 10 passages in mice, whereas infected mouse serum was obtained after passages 2, 10, and 12.

#### *In vitro* culture of trypomastigotes after the *in vivo* step

Part of the bloodstream trypomastigotes produced by *in vivo* infection of Swiss mice after two passages as described above, were used to infect LLC-MK2 and L6 cells. After four passages in culture, the trypomastigotes were collected.

#### RNA extraction

Tissue culture and bloodstream trypomastigote samples were purified by centrifugation at 400× *g* and washed three times with PBS before total RNA extraction using the RNeasy kit (Qiagen), following manufacturer's recommendations. Only parasite samples containing >90% trypomastigotes were used in this study. Cell cultures were PCR-tested weekly for *Mycoplasma* sp. using genus-specific primers [10]. Only PCR-negative cell cultures were used in the experiments. Negative sera from Swiss mice were also obtained by bleeding of uninfected animals maintained in the same experimental conditions.

### Quality control of total RNA and cDNA synthesis

cDNA samples were generated by reverse transcriptase Super Script II (Invitrogen) reactions using random primers and 3–4 µg total RNA. cDNA quality was tested by PCR using specific primers for aromatic L-alpha-hydroxy acid dehydrogenase (AHADH2) (AHADH2_F 5′ CCAAATGTTTCGCCACTCG 3′ and AHADH2_R 5′ CACGCTGCGGAGGGATCTC 3′) and for the DNA repair gene RAD51 (RAD51_F 5′ GGCTGTCAAGGGTATCA 3′ and RAD51_R 5′ AACCACTGCGGATGTAA3′), a gene with low expression in trypomastigotes [11]. Approximately 200 ng of each cDNA or 250 ng of total RNA (negative control) were used in PCR reactions, with 200 µM dNTPs, 2 µM primers, and 1.25 U *Taq* DNA polymerase in 50 mM KCl, 10 mM Tris-HCl pH 8.4, 1% X-100 Triton, and 1.5 mM MgCl_2_. To ensure that the samples were not contaminated with genomic DNA, total RNA samples were tested by PCR for the *T. cruzi* gene AHADH2 using the primers described above. DNA contaminated samples were treated with DNase I (Fermentas), following manufacturer's recommendations, and submitted to RNA clean-up using the RNeasy kit (Qiagen). Treated samples were also PCR-tested. PCR amplification was observed in agarose ethidium bromide or polyacrilamid silver stained gels.

### Construction and sequencing of expression libraries

To construct expression libraries, several primer combinations were tested *in silico* by electronic PCR (e-PCR) (http://www.ncbi.nlm.nih.gov/projects/e-pcr/) and experimentally to amplify the majority of the MASP genes. A semi-nested PCR was set up using the following primer combinations: SL 5′ AACGCTATTATTGATACAGTTTCTGTACTATATTG 3′ for the 35 pb spliced leader region and 3′UTR1 (reverse) 5′ GTGTGCTTCGTGGGGTGAGGTG 3′ for the 3′UTR in the first reaction and SL and 3′UTR2 5′ CTCACTCTCACGCGGCCACCACCACCG 3′ also for 3′UTR (more internal localization) in the second reaction. The semi-nested RT-PCR reactions with these primers were performed with 2 µL of amplified product of the first reaction or 400 ng of the cDNA with 200 µM dNTPs, 2 µM of primers, and 3.75 U High Fidelity *Taq* DNA polymerase (Invitrogen). The amplicons between 500 and 2,000 bp of the second reaction were cloned in pGEM-T (Promega), and at least 96 ampicillin-resistant clones were selected and cultured. After plasmid extraction, a PCR of each clone was performed using primers for the insert flanking regions M13F (forward) 5′ CGCCAGGGTTTTCCCAGTCACGAC 3′ and M13R (reverse) 5′ TCACACAGGAAACAGCTATGAC 3′. Amplified products were precipitated with 20% polyethylene glycol 8000 and 2.5 M NaCl and submitted to sequencing at one end in the *ABI Prism 3730xl DNA Analyser* (Applied Biosystems) by Macrogen Inc (Korea). The libraries constructed for each trypomastigote sample are presented in Table 1. The EST sequences have been deposited in the GenBank database under Accession Number JK743993 - JK744782.

**Table 1:** Libraries codes of the trypomastigote samples.

Library code	Trypomastigote production
Tryp.0.llcmk2	Tissue culture trypomastigotes prior to infection of the two cell types
Tryp.14p.llcmk2	Tissue culture trypomastigotes derived from LLC-MK2 cells after 14 passages
Tryp.14p.l6	Tissue culture trypomastigotes derived from L6 cells after 14 passages
BloodTryp.2p	Bloodstream trypomastigotes after 2 passages
BloodTryp.10p	Bloodstream trypomastigotes after 10 passages
Tryp.4p.llcmk2	Tissue culture trypomastigotes after *in vivo* step derived from LLC-MK2 cells after 4 passages
Tryp.4p.l6	Tissue culture trypomastigotes after *in vivo* step derived from L6 cells after 4 passages

### Sequence and hierarchical cluster analyses

Sequences from the expression libraries were processed by an in-house pipeline. The EST sequences were processed by the Phred algorithm and then filtered after a cross-match with the vector sequence (pGEM-T- Promega). The MASP genes were identified using BLASTN algorithm against two *T. cruzi* sequence databases: one with all *T. cruzi* predicted features of the genome (coding sequences, retroelements and structural RNAs), and the other with *T. cruzi* contigs. The e-value (expected value) cutoff of the BLASTN searches used was 10^−10^, and the minimum identity between the ESTs and the database entries was 70%. The uncertainty of the hierarchical cluster analysis of all expression libraries was calculated using the Pvclust package [12] using the R software platform [13]. Pvclust calculates probability values (p-values) for each cluster using bootstrap re-sampling techniques. AU (approximately unbiased) p-value was used, which is calculated by multi-scale bootstrap re-sampling and has superiority in bias over BP (bootstrap probability) according to the authors [12].

### Representation of MASP genes and transcripts in multidimentional scaling plots

MASP sequence variability and expression profile were visualized in a multidimentional scaling plot (MDS). To this end, we calculated the pairwise distance of all 1,377 annotated MASP genes and generated the distance matrixes using the package PHYLIP [14]–[15]. To provide a visual representation of the distance matrix, we used an MDS plot with two dimensions (2D). The k-means method [16] was used to define six clusters or subgroups. Each dot in the MDS represents a MASP member and its graphic localization, the sequence similarity among the genes. The expressed MASP genes were plotted in the MDS graphics, where the size of the dots represents the frequency of the MASP genes in the library and the colors represent their group classification. The databases generated by the e-PCR predictions were also plotted in the MDS graphics. The MDS, hierarchical clustering, statistical analyses, and graphing were performed using the R software platform [13].

### qRT-PCR

The genes targeted for Real Time PCR (qRT-PCR) were selected after comparative analysis of the expression libraries. Primers were designed using Allele ID 7 (Premier Biosoft, demo version), and NCBI *T. cruzi* database BLAST searches were performed to exclude primers with cross homology with other MASP members and other *T. cruzi* genes. The design also included the template's secondary structure test at 60°C. The MASP complete genes analyzed by qRT-PCR were: MASP2 (Tc00.1047053510359.460), MASP4 (Tc00.1047053508541.110), MASP14 (Tc00.1047053504039.230), MASP16 (Tc00.1047053510693.190), MASP23 (Tc00.1047053511089.19), and MASP27 (Tc00.1047053506615.100). The primer sequences used in the analysis for each MASP gene are listed in Table S1. Reactions in triplicate were prepared with 1 µM forward and reverse primers, SYBR Green Supermix (Applied Biosystems), and each of the diluted template cDNAs (1∶4 in DNAse free water) and were performed using cycling conditions as recommended by the manufacturer (Applied Biosystem). Standard curves were used for the calculation of relative quantity (Rq) values of each sample for each target. qRT-PCRs for MSH2 and RAD51 genes (Table S1) were performed, and the average value between them was used to normalize the MASP gene results. The results were analyzed by a one-way ANOVA test, and graphics were constructed in GraphPad Prism 5.0 (GraphPad Inc.).

### MASP epitope prediction, synthesis of SPOT peptide arrays, and immunoblotting

MASP predicted protein sequences derived from expressed genes were submitted to B-cell linear epitope prediction using the BepiPred algorithm [17], and the output was parsed by an in-house PERL script to select 15-mer amino acid peptides whose prediction score according to their quality as an epitope was >1.3. One or two peptides from specific MASP genes identified in the expression libraries were selected to be synthesized in pre-activated cellulose membranes according to the SPOT synthesis technique [18]. The SPOT synthesis was employed using a method for the preparation of approximately 5 nmol of immobilized peptides. The assembly of the peptides was performed utilizing the previously described Fmoc-chemistry [18]. Briefly, 0.5 mM of each activated Fmoc (9-fluorenylmethoxycarbonyl) amino acid was automatically spotted on pre-activated membranes using the MultiPep SPOT synthesizer (Intavis AG). Each cycle of amino acid coupling was followed by a 10% acetic anhydride blocking and deprotection of Fmoc amino acids by adding 25% 4-methyl piperidine. The coupling and deprotection of Fmoc amino acids were confirmed after each cycle by staining the membrane with 2% bromophenol blue. After the synthesis, the side chain deprotection was performed by adding a 25∶25∶1.5∶1 solution of trifluoroacetic acid, dichloromethane, triisopropylsilane and water. The side-chain deprotection was also confirmed by staining with 2% bromophenol blue. The synthesized peptides are listed in Table S2. Membranes were blocked with 5% BSA and 4% sucrose in PBS overnight and incubated for 1.5 hours with pools of diluted mice sera (1∶500 for IgG or 1∶5,000 for IgM) in blocking solution. After washing three times for 10 minutes in PBS-T (PBS; 0.1% Tween 20), membranes were incubated for 1.5 hours with secondary HRP-conjugated anti-mouse IgM or IgG antibody (Sigma-Aldrich), diluted 1∶2,000 in blocking solution. After a second washing, membranes were revealed by *ECL Plus Western blotting* (GE Healthcare), in the Gel Logic 1500 Imaging system (KODAK). Synthetic peptides corresponding to epitopes of a trans-sialidase [19] and L7A ribosomal protein [20] were included in the experiments as positive controls. As negative controls, membranes were submitted to the same experimental conditions using sera of uninfected Swiss mice. Densitometry measurements and analysis of each peptide were performed using Image Master Platinum (GE), and the relative intensity ratio (RI) cutoff for positivity was determined as 2.0. Reactive spots in the positive blottings (using the infected mouse serum pool) were only selected for analysis when not reactive in the negative blotting. Graphics were constructed in GraphPad Prism 5.0 (GraphPad Inc.).

### ELISA

Seven peptides with the highest RI values and a peptide derived from trans-sialidase SAPA (shed acute phase antigen) [21] (Table S3) were submitted to soluble synthesis (Peptide 2.0) and ELISA experiments. Flexible ELISA polyvinylchloride plates (BD Falcon) were sensitized with 2 µg of soluble peptides or trypomastigote extract in water at 37°C overnight. After blocking with 2.5% BSA in PBS for 2 hours at 37°C, the plates were incubated with sera from uninfected and infected mice (dilution 1∶100) for 1.5 hours at 37°C. After washing in 0.05% Tween 20-PBS, the plates were incubated with secondary antibodies anti-mouse IgM or IgG (dilution 1∶2000; Sigma). After several washes in PBS-0.05% Tween 20, the plates were revealed with OPD (o-phenylenediamine; Sigma), in citric acid buffer (50 mM Na_2_HPO_4_, 27 mM, citric acid, pH 5.0) and hydrogen peroxide and read at 492 nm. The reactivity of the trypomastigote extract was used in the normalization of ELISA results using the peptides. The results were analyzed by one-way ANOVA test and graphics were constructed with GraphPad Prism 5.0 (GraphPad Inc.). Trypomastigote total extracts were obtained by ultrasound lysis of purified and PBS-washed parasite. Protein quantification was determined by the BCA™ Protein Assay Kit (Pierce).

### Avidity ELISA

Low-affinity antibodies were eluted by adding an incubation step with 6 M urea for 5 minutes at room temperature after the mouse serum incubation. Avidity index was expressed as (mean OD of urea-treated sera/mean OD urea-untreated sera)×100%. Affinity indexes <40% or >40% were considered low and intermediate affinity levels, respectively [22]. The three mouse serum pools (after 2, 10, and 12 passages) were tested in triplicate.

### Invasion assay

Trypomastigotes derived from L6 cells after 17 passages were purified by centrifugation at 400× *g* followed by incubation at 37°C for 4 h to allow motile trypomastigotes to swim up. The trypomastigotes were then used to infect LLC-MK2 and L6 cells as follows. LLC-MK2 or L6 cells ressuspended in RPMI supplemented with 10% FBS were plated (4×10^4^ cells/well) in 24-well plates containing coverslips and incubated at 37°C and 5% CO_2_ for 36 hours prior to infection. Infection was performed by exposing cells to purified trypomastigotes for 30 min at 37°C at a multiplicity of infection (MOI) of 50. Cells were then washed five times with PBS to remove extracellular parasites and fixed with 4% (wt/vol) paraformaldehyde/PBS overnight at 4°C. After fixation, coverslips with attached cells were washed three times in PBS, incubated for 20 min with PBS containing 2% BSA, and processed for an inside/outside immunofluorescence invasion assay using an anti-*T. cruzi* rabbit polyclonal antibody and a secondary anti-rabbit IgG antibody conjugated with Alexa Fluor 546 (Life technologies) according to a previous protocol [23]. In this step, all extracellular, non-washed parasites were stained. Coverslips were then washed twice with PBS, incubated with 10 g/ml DAPI (4′,6′-diamidino-2-phenylindole; Sigma) in PBS for 2 min, washed three times in PBS and mounted on slides using a fluorescence mounting solution containing 1 mg/ml of PPD (p-phenylenediamine) in Glycerol/Tris-HCl. At least 250 cells (10 fields) were analyzed per coverslip in triplicate, and invasion rates were calculated as the number of intracellular parasites/100 host cells. Graphs were plotted using GraphPad Prism 5.0 (GraphPad Inc.) and statistically significant differences were determined using Student's *t* test.

### Ethics statement

All animal procedures were approved by the animal-care ethics committee of the Federal University of Minas Gerais (Protocol # 232/2009) and were performed under the guidelines from COBEA (Brazilian College of Animal Experimentation) and strictly followed the Brazilian law for “Procedures for the Scientific Use of Animals" (11.794/2008).

## Results

### MASP subgroups and library construction

Prior to investigating the MASP expression profile, we performed sequence clustering analysis of the family to identify subgroups, whose expression profiles were then analyzed. To this end, we performed pairwise alignments of the coding sequences of all MASP genes, resulting in a distance matrix that was used to generate a multidimensional scaling (MDS) plot (Figure S1). The k-means method was used to define six clusters or subgroups (Figure S1A) (Table S4). Due to the extensive sequence variability of the MASP family, we performed a series of electronic PCR analyses to select the primers suitable to amplify most MASP transcripts. These analyses suggest that most of the MASP complete genes (77.8% or the equivalent to 630 genes) and eventually those derived from pseudogenes (55.9%, 317 members) could be amplified in a semi-nested RT-PCR using primers for the spliced leader sequence (SL) and MASP 3′UTR (3′UTR1 and 3′UTR2) (Figure S1B). The SL primer was used in both reactions of the semi-nested RT-PCR to guarantee that the amplified transcripts were mature, whereas the 3′UTR1 and 3′UTR2 primers were derived from the MASP 3′UTR, which is the most conserved region of MASP transcripts [5]. In addition, because of the mosaic structure of MASP genes having shared fragments among the members [4], this combination of primers has the advantage of generating amplification products containing the entire MASP coding region and therefore would allow an unequivocal identification of the expressed genes. Using these primers, five of the six different MASP subgroups can be amplified (Figure S1).

### MASP expression profile in culture and bloodstream trypomastigotes

**Figure 2:**
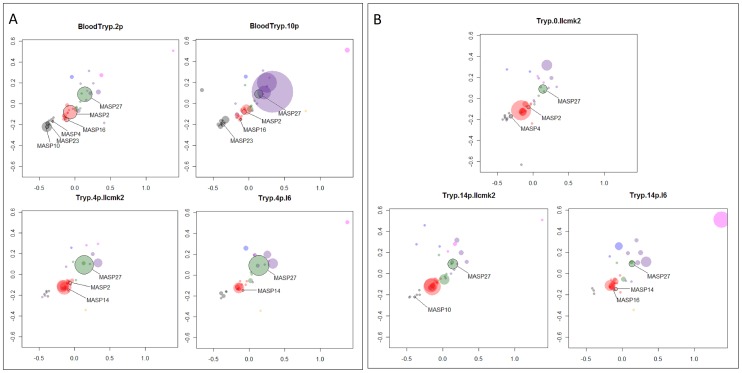
MDS distribution of sequences from the MASP expression libraries. **Figure 2A**: MDS distribution of sequences from the MASP expression libraries of bloodstream forms from sequential passages and of culture trypomastigotes after the *in vivo* step. **Figure 2B**: MDS distribution of sequences from the MASP expression libraries of culture trypomastigotes derived from distinct cell types. Black bordered genes: MASP complete genes analyzed by qRT-PCR. Tryp.0.llcmk2: culture trypomastigotes prior to infection of the two cell types; Tryp.14p.llcmk2: culture trypomastigotes derived from LLC-MK2 cells after 14 passages; Tryp.14p.l6: culture trypomastigotes derived from L6 cells after 14 passages; BloodTryp.2p: bloodstream forms after 2 passages; BloodTryp.10p: bloodstream forms after 10 passages; Tryp.4p.llcmk2: culture trypomastigotes after *in vivo* step derived from LLC-MK2 cells after 4 passages; Tryp.4p.l6: culture trypomastigotes after *in vivo* step derived from L6 cells after 4 passages.

We hypothesized that the large repertoire of MASP peptides may contribute to ability of *T. cruzi* to infect and/or survive within several host-cell types and/or participate in host immune evasion mechanisms [5]. To begin investigating this hypothesis, we constructed seven expression libraries from tissue culture trypomastigotes derived from two cell types (epithelial cells and myoblasts), and from bloodstream trypomastigotes recovered after sequential passages in mice (Table 1). For all libraries, the amplification profile by semi-nested RT-PCR presented a smear in both reactions (Figure S2), indicating the co-expression of several MASP transcripts with different lengths. Fragments ranging from 500 to 2,000 bp were cloned into pGEM-T vector (Promega) and a total of 960 clones were sequenced. Based on the percentages of valid sequences, ranging from 76.4% to 90.6%, the libraries were considered to be of good quality (Table 2). The *T. cruzi* genes corresponding to the best hit of each valid EST sequence are shown in the Table S5. We retrieved a total of 94 MASP complete genes by analyzing the content of the libraries altogether, even though the number of sampled transcripts per library was restricted to 20 to 32 genes. Although the proportion of sequences with non-MASP best hit ranged from 50% to 83.9%, the majority of these genes are short hypothetical proteins that appear to be unreal because they were predicted within the MASP 3′UTR, which contains the annealing sites of the reverse primers used to construct the libraries. Other non-MASP hits include chimeric sequences containing the MASP C-terminal coding sequence and the downstream MASP 3′UTR and TcMUC mucin genes and retroelements followed by fragments of the MASP 3′UTR. We also detected the transcription of pseudogenes, ranging from 4.8% to 37.8% of the MASP hits in all the libraries. Because the library of bloodstream trypomastigotes after 10 passages (Blood.Tryp.10p) presented a higher proportion of pseudogenes (37.8% versus an average of 12.3% for all other libraries), a larger number of sequences from this library was analyzed (Table 2). We analyzed 96 more sequences of the Blood.Tryp.10p expression library than of the other libraries, and the number of MASP genes sampled in this library was similar to those of the other libraries. We mapped the MASP cDNAs in the MDS distribution to represent a visual analysis of the expressed members. A transparency was applied to the dots so that differences in their size would represent differences in the level of MASP expression (Figure 2).

**Table 2:** Sequencing results of the MASP libraries of culture and bloodstream trypomastigotes.

Libary code	Clones sequenced	Valid sequences	MASP hits*	MASP Complete gene sequences**	MASP Pseudogene sequences**	Unique MASP complete genes	Unique MASP pseudogenes
**Tryp.0.llcmk2**	96	86; 89.6%	62; 72,1%	59	3	26	3
**Tryp.14p.llcmk2**	144	116; 80.6%	77; 66,4%	69	8	28	6
**Tryp.14p.l6**	144	110; 76.4%	57; 51,8%	47	10	20	5
**Tryp.4p.llcmk2**	144	110; 76.4%	76; 69,1%	63	13	23	8
**Tryp.4p.l6**	144	120; 83.9%	60; 50%	52	8	21	2
**BloodTryp.2p**	96	87; 90.6%	73; 83,9%	65	8	28	6
**BloodTryp.10p**	192	161; 83.9%	90; 55,9%	56	34	32	5
**Total**	960	790	495	411	84	94***	21***

* The majority of non-MASP hits are derived from short hypothetical proteins that seem unreal since were predicted within MASP 3′UTR.

** Redundant matches taken into account.

*** Excluding redundant matches among the different libraries.

Valid sequences: quality tested sequences used in the analysis; MASP hits: valid sequences identified as MASP genes; Tryp.0.llcmk2: culture trypomastigotes prior to infection of the two cell types; Tryp.14p.llcmk2: culture trypomastigotes derived from LLC-MK2 cells after 14 passages; Tryp.14p.l6: culture trypomastigotes derived from L6 cells after 14 passages; BloodTryp.2p: bloodstream forms after 2 passages; BloodTryp.10p: bloodstream trypomastigotes after 10 passages; Tryp.4p.llcmk2: culture trypomastigotes after *in vivo* step derived from LLC-MK2 cells after 4 passages; Tryp.4p.l6: culture trypomastigotes after *in vivo* step derived from L6 cells after 4 passages.

**Figure 3:**
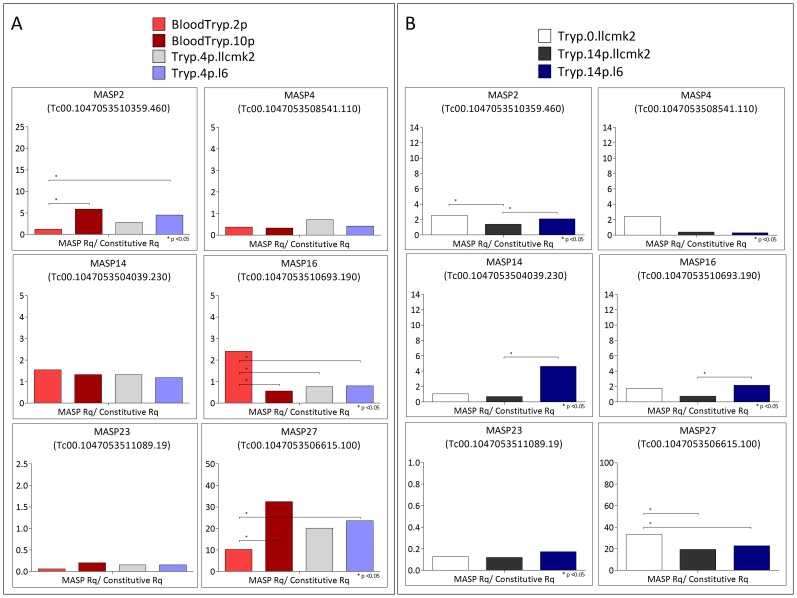
Expression analysis by qRT-PCR of selected MASP genes. Relative quantity (Rq) calculations were based on specific standard curves for each MASP gene. Rq values of each cDNA sample (MASP Rq) were normalized with the average of two constitutive genes MSH2 (MSH2 Rq) and RAD51 (RAD51 Rq). **Figure 3A**: Expression analysis by qRT-PCR of six MASP genes in bloodstream forms from sequential passages and of culture trypomastigotes after the *in vivo* step. **Figure 3B**: Expression analysis by qRT-PCR of six MASP genes in culture trypomastigotes derived from distinct cell types. Tryp.0.llcmk2: culture trypomastigotes prior to infection of the two cell types; Tryp.14p.llcmk2: culture trypomastigotes derived from LLC-MK2 cells after 14 passages; Tryp.14p.l6: culture trypomastigotes derived from L6 cells after 14 passages; BloodTryp.2p: bloodstream forms after 2 passages; BloodTryp.10p: bloodstream forms after 10 passages; Tryp.4p.llcmk2: culture trypomastigotes after *in vivo* step derived from LLC-MK2 cells after 4 passages; Tryp.4p.l6: culture trypomastigotes after *in vivo* step derived from L6 cells after 4 passages.

In a previous study, we had identified several MASP transcripts in a cDNA library constructed from tissue culture trypomastigotes derived from Vero cells [5]. Here, we investigated whether MASP is also expressed in bloodstream trypomastigotes during the acute phase of experimental infection. In addition, we compared two expression libraries constructed from bloodstream trypomastigote forms after sequential passages in mice. As shown in Figure 2A, bloodstream trypomastigotes derived from a given passage co-express MASP genes belonging to all five different groups, indicating a broad expression of different MASP genes. MASP expressed genes for which we could design specific primers were analyzed by qRT-PCR. Significant differential expression was observed by qRT-PCR for MASP2, MASP16, and MASP27 between bloodstream trypomastigotes from sequential passages (Figure 3A). MASP2 and MASP27 were significantly more expressed in bloodstream trypomastigotes after 10 passages in mice compared to trypomastigotes after two passages. In contrast, MASP16 was significantly more expressed in bloodstream forms after two passages in mice. In addition to the temporal changes in the expression of a given gene after sequential passages in mice, we also found that the level of expression of distinct MASP transcripts varies significantly in the trypomastigote population. For instance, MASP27 is 100 times more expressed than MASP 23 in all libraries (Figure 3). These results indicate that the expression profile of distinct MASP genes is heterogeneous and may vary after sequential passages in mice.

To investigate whether tissue culture trypomastigotes had a distinct MASP expression profile compared with the bloodstream forms, part of the trypomastigote population collected after two passages in mice was used to infect myoblast (L6) and epithelial cells (LLC-MK2), and after 4 passages in culture, the RNA was extracted for library construction. Both libraries, Tryp.4p.llcmk2 and Tryp.4p.l6, showed a very similar pattern of expression (Figure 2A). In fact, no significant difference in gene expression between these two libraries was observed for those genes analyzed by qRT-PCR (Figure 3A). In contrast, we detected differences in the expression profile of bloodstream forms after two passages (BloodTryp.2p) compared with both Tryp.4p.llcmk2 and Tryp.4p.l6 libraries (Figure 2A). This was confirmed by qRT-PCR: MASP2 and MASP27 were more expressed in Tryp.4p.l6 compared to the BloodTryp2 library, whereas MASP16 was more expressed in bloodstream forms after two passages compared with L6- and LLC-MK2-derived trypomastigotes after four passages (Figure 3A).

Because we did not detect significant changes in MASP expression when comparing trypomastigotes after four passages in the two types of host cells (myoblast and LLC-MK2 cells), we decided to analyze the MASP expression profile after a larger number of passages in these two types of tissue culture cells (14 passages). Similar to what was observed in the other libraries, in both Tryp.14p.llcmk2 and Tryp.14p.l6 libraries, we also detected co-expression of MASP genes in five different groups. Furthermore, MASP27 is one of the most represented genes in the sequenced clones, while MASP23 was not sampled in these libraries (Figure 2B). This data were validated by qRT-PCR since we detected remarkably high levels of expression of MASP27 compared to the other analyzed genes, while MASP23 was approximately 100 times less expressed in all libraries (Figure 3B). More importantly, more notable differences in MASP expression in trypomastigotes derived from both host cells were observed after 14 passages compared to the expression profile after 4 passages. We observed by qRT-PCR that MASP2, MASP14, and MASP16, belonging to the Red subgroup, were more expressed in L6-derived trypomastigotes compared to LLC-MK2-derived trypomastigotes after 14 passages (Figure 3B). Whether these specfiic MASP members are implicated in trypomastigote invasion, replication, and/or survival within L6 cells remain to be investigated. Nevertheless, by performing invasion assay, we confirmed an association between the MASP profile and the infectivity of L6-derived trypomastigotes (Figure S3). Specifically, we evaluated the rate of invasion of L6 and LLC-MK2 cells by the same population of trypomastigotes that were maintained for 17 consecutive passages in L6 cells. We found a higher rate of invasion of L6 cells compared with LLC-MK2, reinforcing our findings and suggesting that successive passages of trypomastigotes in a given host cell may configure a specific expression profile that optimizes the rate of invasion.

**Figure 4:**
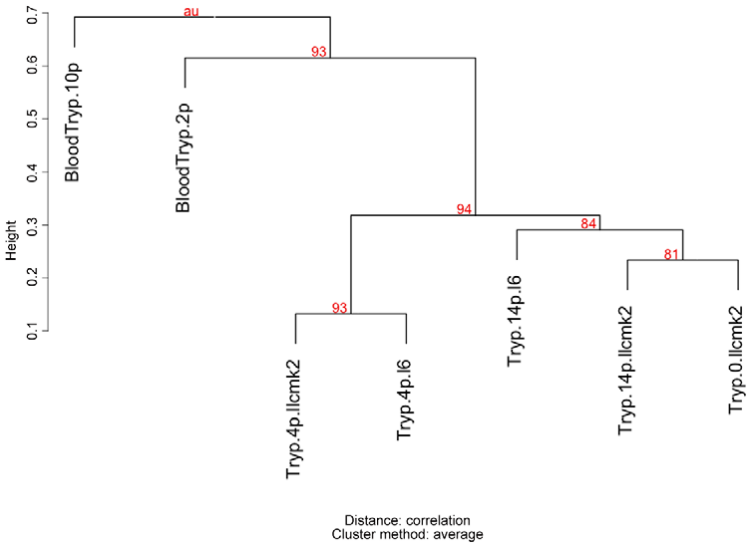
Dendrogram of hierarchical analysis of MASP expression libraries. The uncertainty of the hierarchical cluster analysis of all expression libraries was calculated using Pvclust package [10]. AU values (in red): approximately unbiased p-value.

The differences observed by qRT-PCR are in agreement with the hierarchical clustering analysis of all expression libraries using the *pvclust* package (Figure 4). The dendrogram derived from the *pvclust* analysis shows that, after four passages, tissue culture trypomastigotes derived from either L6 or LLC-MK2 cells are very similar. In contrast, tissue culture trypomastigotes libraries are distantly clustered from bloodstream trypomastigote libraries. Taken together, these results indicate that tissue culture and *in vivo* infection may selectively configure a distinct MASP expression profile in trypomastigotes.

### MASP antigenic profile in the acute phase of the experimental infection

**Figure 5:**
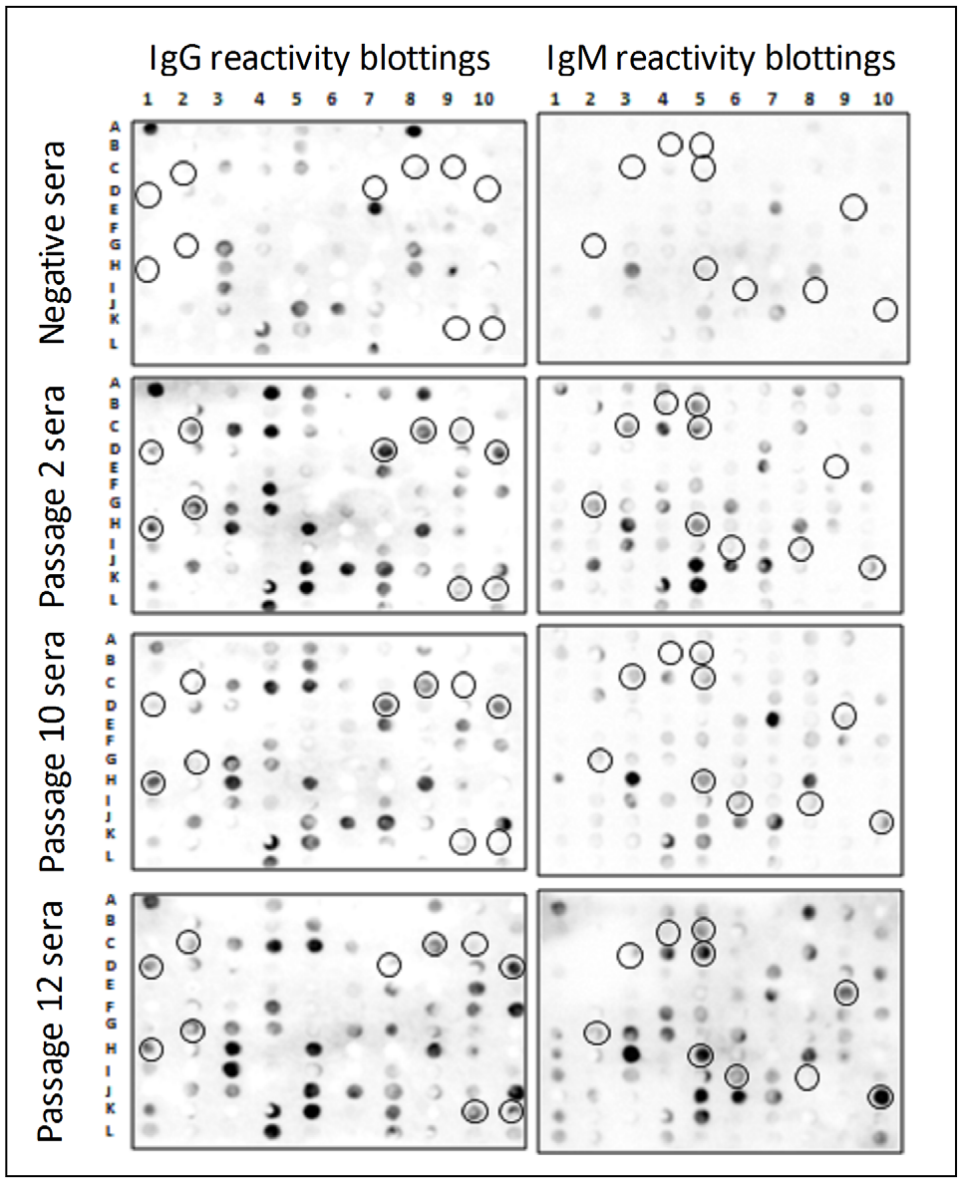
Screenings of MASP B-cell epitopes using SPOT peptide arrays. Blotting representative images using sera pools (n = 10) after 2, 10, and 12 passages in mice of CL-Bnener *T. cruzi*; predicted peptides were covalently synthesized in pre-activated cellulose membranes according to the SPOT synthesis technique (Frank *et al.*, 1992); circled spots: peptides with the highest Rd values.

To investigate the MASP antigenic profile in the acute phase of the experimental infection, we performed B-cell linear epitope prediction on the MASP proteins derived from expressed members using the Bepipred algorithm [17]. Only predicted epitopes exclusive for MASP proteins were selected. A total of 110 peptides for 64 MASP expressed genes, from a total of 94 genes identified in the expression libraries, are member specific and were analyzed by immunoblotting. We found that 74 to 88% of the analyzed MASP members were recognized by sera of acutely infected mice, having at least one reactive peptide (RI>2.0) against one serum pool (Figure 5). Additionally, 21 to 33% of the MASP members were recognized by all three serum samples. The remaining peptides were not reactive or were excluded from the analysis after the normalization step with sera of uninfected mice.

**Figure 6:**
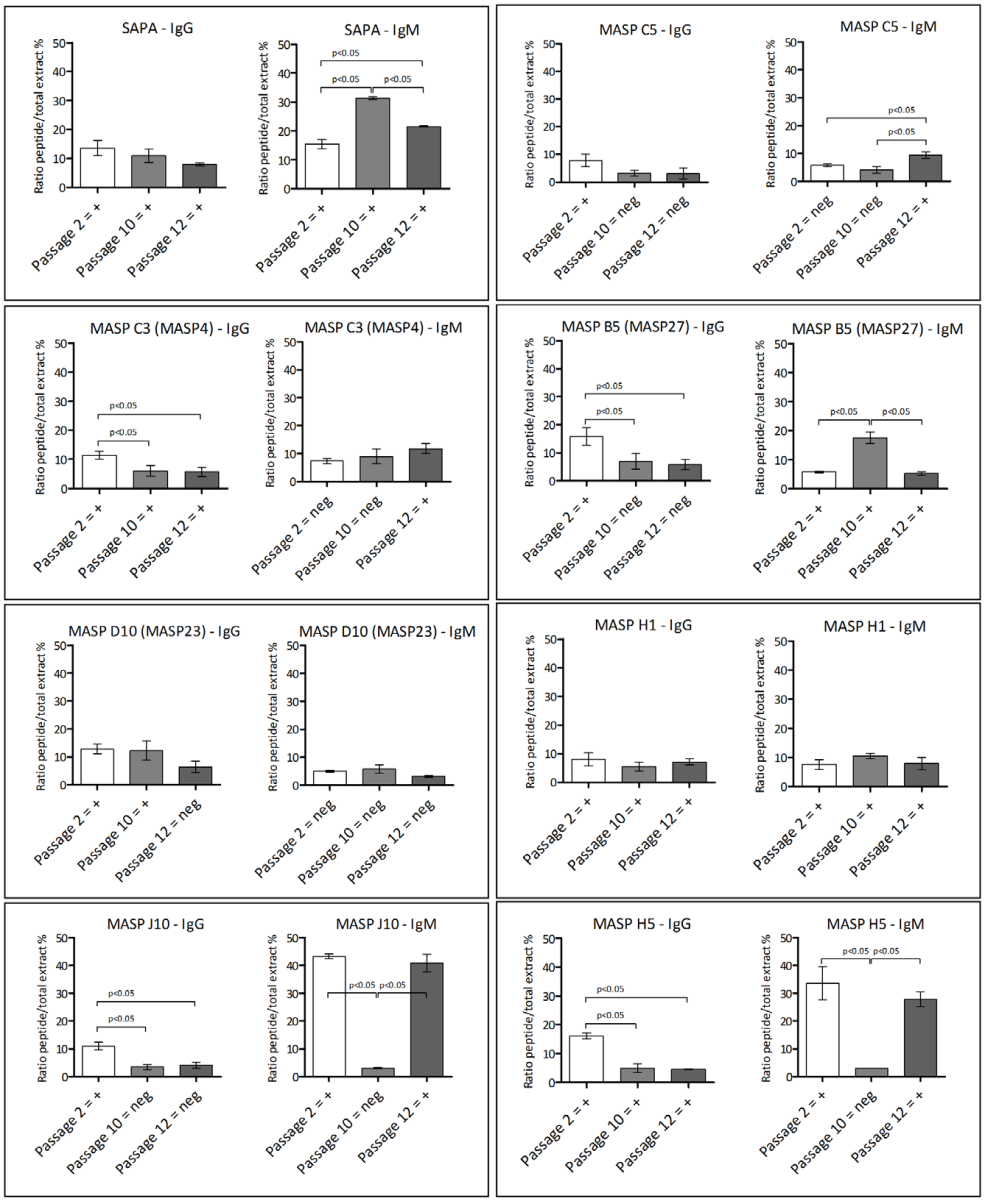
MASP peptide ELISA. Seven MASP peptides and SAPA were submitted to soluble synthesis (Peptide 2.0) and ELISA experiments. Flexible ELISA plates (Falcon) were sensitized with 2 µg of soluble peptides, incubated with uninfected and infected mouse serum pools from three passages, IgG or IgM secondary antibodies, and revealed with OPD solution. Absorbance values were normalized with values resulting from ELISA of each passage against total trypomastigote extract.

Following the peptide screenings on SPOT arrays, seven peptides with high RI values were submitted for soluble synthesis to be used in ELISA experiments (Table S3). As expected, IgM and IgG antibodies from infected mice were reactive against all MASP peptides and also against SAPA, a known *T. cruzi* epitope derived from a repetitive region of the trans-sialidase enzyme [21] (Figure 6). MASP genes with low expression levels had reactive peptides, such as peptide D10, derived from MASP23. However, it is important to emphasize that mRNA and protein levels in *T. cruzi* are not always linearly associated, and therefore we cannot assume that the level of expression of the MASP23 protein is also low. A variable level of reactivity against MASP peptides was also observed between the sequential passages in mice. Furthermore, variable levels of recognition by the two immunoglobulin types (IgG and IgM) of each peptide were observed. High levels of IgM were observed against peptide B5, derived from MASP27, after ten passages in mice. It is worth noting that, as mentioned before, MASP27 had the highest expression levels in all cDNA libraries. High levels of IgM were also observed against the peptides H5 and J10 after two and 12 passages in mice. Both peptides are derived from MASP genes with low expression levels. The other peptides tested by ELISA that were derived from genes expressed at low levels, displayed a distinct level of antibody recognition. SAPA reactivity by IgM had high values, and the results also indicated that there was a differential recognition by IgM antibodies between the sequential passages in mice. The affinity levels of the IgG and IgM antibodies against MASP peptides were also measured (Figure S4). The IgG antibodies against all tested MASP peptides presented intermediate affinity, ranging from 52 and 58.3%, which were higher levels than that of the affinity of antibodies against SAPA (40%). In contrast, IgM antibodies against MASP peptides presented variable levels of affinity. IgM antibodies against C5 peptides presented intermediate affinity (54.9%), whereas antibodies against MASP peptides C3, B5, D10, H1, and SAPA presented low affinity, ranging from 17.8 to 33.2%. The distinct antibody affinity levels are most likely related to differences in peptide composition rather than to the total antibody levels against the peptides. For instance, antibodies against the peptides C5 had the highest affinity index, despite the fact that the total antibody against this peptide had the lowest level (Figure S4).

Overall, these results showed that different members of the MASP family are expressed during acute *T. cruzi* infection and constitute parasite antigens recognized by IgG and IgM antibodies. The results also indicated that distinct MASP peptides could trigger different antibody responses and that the antibody level against a given peptide may vary after sequential passages in mice.

## Discussion

A key *T. cruzi* strategy to survive in a mammalian host is its ability to actively invade a wide variety of non-phagocytic host cells. The acute phase of Chagas disease is characterized by intense parasitaemia and tissue parasitism involving the infection of heart, skeletal and smooth muscle cells, as well as liver, fat, and brain cells [24]–[27]. Furthermore, during the chronic phase, there are several reports on differences of *T. cruzi* tropism to host tissue, which is associated with the pathogenesis of Chagas disease [28]–[31]. Therefore, an important aspect to understanding *T. cruzi* infection is the identification of molecular components of both parasite and host cells that play a role in the infection of a broad range of cell types. In this regard, several studies have investigated changes in gene expression during *T. cruzi* infection. However, these studies have focused mainly on the modulation of gene expression of the host cells [32]–[40]. Although several trypomastigote surface proteins have been implicated in host-cell recognition/invasion [41]–[46], so far there is no clear association between a *T. cruzi* expression profile and its ability to invade/proliferate in a given host cell.

In our previous work on the molecular characterization of the MASP family [5], we speculated that these proteins may be involved in host–parasite interactions because of their surface localization on the infective circulating trypomastigote forms. Moreover, the MASP family is highly polymorphic and can be secreted by the parasite, thus contributing to a large *T. cruzi* polypeptide repertoire that could be exposed to the host cells and the host's immune system [5]. In fact, it has been shown recently that a MASP protein is able to induce endocytosis in Vero cells [47], a process whereby the trypomastigote forms of the parasite actively invade host cells [48]. As an attempt to investigate whether MASP members could be implicated in interactions with specific cell types, we investigate the MASP expression profile in trypomastigotes derived from epithelial (LLC-MK2) and myoblast (L6) cell lines. We selected these cell types because LLC-MK2 is widely used for maintaining *T. cruzi* in *in vitro* culture, whereas myoblasts give raise to muscle cells, which are target by *T. cruzi* during acute and chronic phase of Chagas disease [26], [49]. We did not detected significant changes in the MASP expression profile between these two host cells after 4 passages in tissue culture. However, differential expression of MASP genes were detected by sequencing and by qRT-PCR analyses between trypomastigote forms derived from these two host cells after a larger number of tissue culture passages: MASP2, MASP14 and MASP16 were significantly more expressed in myoblasts compared to epithelial cells after 14 passages (Figure 3B). This is an indirect evidence that different MASP genes may be implicated in the interaction with distinct cell types. As indicated before, it was recently demonstrated that one MASP member (named MASP52, Tc00.1047053504239.220) is secreted by trypomastigote forms upon contact with non-phagocytic Vero cells and is able to induce endocytosis [47]. This MASP gene was not sampled in our sequenced clones. Nevertheless, an association between the selection of a MASP profile and the infectivity of L6-derived parasites is suggested by the invasion assay experiment (Figure S3). Whether peptides derived from MASP2, MASP14 and MASP16 are involved in myoblast recognition/invasion and/or parasite proliferation/survival within myoblast cells remains to be investigated.

We have also investigated whether the transition from *in vivo* to *in vitro* infection would affect the MASP expression profile. To this end, the same trypomastigote population that was recovered from passage 2 in mice was used to infect myoblast and epithelial cells, and after four passages in each cell type, the MASP expression profile was analyzed. We found noticeable differences in the MASP family expression profile when tissue-culture and bloodstream forms were compared (Figures 2A, 3A and 4). Significant differential expression of the genes MASP2, MASP16 and MASP27 was observed by qRT-PCR in tissue-culture derived trypomastigotes after four passages (Tryp.4p.llcmk2 and Tryp.4p.l6) compared to bloodstream trypomastigotes after two passages (BloodTryp.2p) (Figure 3A). These observations suggest that selective pressure driven by tissue culture or *in vivo* infection may induce distinct MASP expression profiles. Differences in infections using bloodstream- and tissue culture-derived trypomastigotes have already been reported. Specifically, it has been shown that the rate of *in vitro* infection of distinct host cell types does not correlate with the level of parasitaemia of experimentally infected mice [50]. We also found a heterogeneous MASP expression profile when bloodstream trypomastigotes recovered from mice after 2 and 10 passages were compared: MASP2 and MASP16 were significantly more expressed in bloodstream forms after 10 passages compared with bloodstream forms from passage 2, and the inverse was observed for MASP16 (Figure 3A). In addition, a large number of MASP pseudogenes were expressed in bloodstream forms after 10 passages, which was not observed for any other library (Table 2). Sequencing a larger number of clones of this library did not significantly change this profile. Pseudogenes may contribute to the diversity of the sequence repertoire through recombination. Indeed, the involvement of pseudogenes in the generation of variant surface glycoprotein (VSG) diversity has already been described in *T. brucei* [51], and it was hypothesized that this mechanism may explain the large number of VSG pseudogenes in the *T. brucei* genome [52]. Whether there is a selective pressure imposed by the vertebrate host immune system to increase the sequence diversity of the MASP family in the circulating trypomastigote forms at the expenses of generating pseudogenes remains to be investigated.

It is well established that several *T. cruzi* surface proteins are co-expressed at a given time in the parasite population, leading to the phenomenon of antigenic variability [53]. Here, we found that, in fact, several MASP genes are co-expressed, although the level of expression of each transcript is very variable in a given library (Figure 3), indicating a heterogeneous expression of the family. This expression profile was also observed in the proteomic study of the trypomastigote form of the Y strain [7]. In this study, 37 unique MASP peptides found in 167 MASP proteins were identified at different expression levels in a parasite population derived from LLC-MK2 host cells. Although the conditions and strains used in both studies were different, it is interesting that two MASP members sampled in our expression libraries from trypomastigotes derived from LLC-MK2 host cell were also represented in this proteomic study (Tc00.1047053508253.10 and Tc00.1047053510163.30). We have previously analyzed the MASP expression in individual trypomastigotes by performing immunofluorescence using non-permeabilized cells and an affinity-purified antibody specific to a MASP subgroup. Only a few trypomastigotes were labeled with the antibody, suggesting that, at least for some MASP proteins, their expression on the surface of trypomastigotes is not uniform in the parasite population [5]. The present study added another layer of complexity to the expression of the MASP family since we detected temporal changes in gene expression of the same gene after sequential passages in mice and also in trypomastigotes derived from epithelial and myoblast cells after a large number of *in vitro* passages (Figures 2 and 3).

How the parasite modulates the expression of MASP genes during the infection is an open question. We did not detect a correlation between chromosomal location of the MASP genes sampled in our cDNA libraries and their expression levels (data not shown), suggesting that there is no apparent bias regarding the chromosomal location of expressed MASP genes. It is well established that the control of gene expression in Trypanosomatids operates almost exclusively at a post-transcription level, primarily mediated by regulatory elements with the 3′UTR of the transcripts that modulate the mRNA stability by means of interactions with regulatory proteins [reviewed in 54]. We have previously shown that the 3′UTR of MASP transcripts is highly conserved among the family members [5] and therefore may not be involved in the differential expression of the distinct MASP genes. Nevertheless, subtle nucleotide differences in these regions and/or alternative polyadenylation sites among the different transcripts may favor or abolish specific interactions with regulatory proteins. How the parasite changes the expression of the same MASP gene under different *in vitro* and *in vivo* conditions is also intriguing. In this case, it is possible that the host cell and/or the host immune system may configure a specific MASP expression profile.

Another possible MASP function that may explain the high level of polymorphism of the family would be its involvement in immune evasion mechanisms. In addition to its extreme polymorphism, localization at the trypomastigote surface, and shedding properties, another MASP feature that reinforces this hypothesis is the large repertoire of distinct repetitive motifs of the MASP proteins [5]. It has been shown that several parasitic repetitive proteins are targets for strong B-cell responses [53], [55]–[56]. In fact, *in silico* predictions performed by our group on the entire MASP proteome suggest the occurrence of a large repertoire of B-cell epitopes in the family (data not shown). In the present study, we validated these predictions by showing that several peptides derived from MASP-expressed members reacted with sera from acutely infected mice (Figures 5 and 6). The antibody recognition of several MASP peptides supports the interaction of the MASP family with the host immune system during acute *T. cruzi* infection.

We have also investigated whether the MASP antigenic profile changes during acute infection. Indeed, variable antigenic profiles between the trypomastigotes isolated from sequential passages in experimentally infected mice were observed by immunoblotting and ELISA (Figures 5 and 6). The MASP family, along with other *T. cruzi* surface proteins, may contribute to the polyclonal lymphocyte activation that leads to hypergammaglobulinemia and the delayed specific humoral immune response, that are characteristic of the acute phase of Chagas disease. These phenomena are suggested to be an immune evasion mechanism [57]–[59]. Polyclonal lymphocyte B activation could scatter the immune response, preventing the development of a specific and neutralizing response against the parasite and its complete elimination. T-cell independent responses may contribute to hypergammaglobulinemia [57]. Indeed, the SAPA repetitive C-terminal region of the trans-sialidase protein was reported to be a T-cell independent B mitogen and inducer of non-specific Ig secretion [56]. It is possible that MASP peptides could mediate both specific T-dependent or unspecific T-independent immune responses, a hypothesis that is partially supported by the differential recognition of MASPs by the two immunoglobulin types (IgM and IgG) and the difference in the antibody affinity levels against each of the synthetic peptides. We speculate that variations in the large repertoire of antigenic peptides derived from the MASP family may contribute to the mechanism of immune evasion during the acute phase of the infection.

This is the first report on the antigenic properties of the MASP family, supported by the description of the antibody recognition of expressed MASP peptides in the acute phase of the experimental infection. MASP expression in bloodstream trypomastigotes is also first described in this study, as well as the differential expression of its members in trypomastigotes derived from distinct host cells and during acute experimental infection. The MASP expression profile is likely to be even more complex than reported here due to the limitations of our approach. The construction of the expression libraries in our investigation was limited by the similarity of the MASP 3′UTRs, and by the limited number of clones sequenced. Nevertheless, this study revealed a much more complex pattern of MASP expression than was previously described [5]. The use of an RNA-*seq* approach to study the transcription of bloodstream, tissue-culture derived parasites and infected host cells will reveal a comprehensive picture of the expression of genes involved in *T. cruzi*–host cell interactions.

## Supporting Information

Figure S1 **Multidimensional scaling (MDS) plot of MASP genes.** Pairwise alignments of the 1,377 MASP genes were performed and the distance matrix was used to generate a multidimensional scaling (MDS) plot. K-means method was used to define the clusters or groups. **A**: MDS distribution of all 1,377 MASP genes; **B**: MDS distribution of the 947 MASP genes amplified by e-PCR allowing 2 gaps and 2 mismatches in the primer annealing sequences. Pseudogenes are shown in purple color.
(TIF)

Figure S2 **Construction of the MASP expression libraries: RNA and cDNA quality controls and semi-nested RT-PCR for MASP representative agarose (A, B, D, and E) or polyacrilamide (C), electrophorese gels.** **A**. 1 to 7: PCR for AHADH2 gene using total RNA as template; 1a, 2a, and 3a: same RNA samples before DNAse I digestion, DNA contaminated. **B**. 1 to 7: PCR for AHADH2 gene using the cDNAs as templates corresponding to each RNA sample; CNRT: PCR using a negative cDNA (with no RNA) as template; **C**. 1 to 7: PCR for RAD51 gene using the cDNAs as templates corresponding to each RNA sample. **D**. 1 to 7: First PCR reaction for MASP family using each cDNA as template, with primers SL and 3′UTR1. **E**. 1 to 7: Second PCR reaction for MASP family using each PCR sample as template, with primers SL and 3′UTR2; gDNA: PCR using 10 ng *T. cruzi* genomic DNA as template; CN: negative control of PCR reaction, with no DNA template.
(TIF)

Figure S3 **Invasion assay.** LLC-MK2 or L6 cells were infected with trypomastigotes derived from L6 cells after 17 passages, fixed, and processed for immunofluorescent detection of intracellular parasites. The data correspond to the mean of triplicates ± SD and were analyzed using the Student's *t* test. The results are representative of one of two experiments that yielded similar results.
(TIF)

Figure S4 **Affinity ELISA of MASP peptides.** After the incubation with sera pool of mice infected with *T. cruzi* after two passages, a single wash step was added to the peptide ELISA protocol with 6 M urea. The results of absorbance were compared to the respective non-washed samples (A and C) in the same experiment. Affinity levels <40% and >40% were considered intermediate and low affinity indexes, respectively (dotted lines). A and B: total reactivity (A) and affinity levels (B) of IgG antibodies against the MASP peptides; C and D: total reactivity (C) and affinity levels (D) of IgM antibodies against the MASP peptides.
(TIF)

Table S1 **List of primers used in the Real Time RT-PCR analysis.**
(DOC)

Table S2 **List of MASP peptides analyzed by immunoblotting.**
(XLS)

Table S3 **List of soluble peptides used in ELISA experiments.**
(DOC)

Table S4 **List of members of each MASP group.**
(XLS)

Table S5 **EST annotation.**
(XLSX)
