# Supplementary figures and images for: The MASP Family of Trypanosoma cruzi: Changes in Gene Expression and Antigenic Profile during the Acute Phase of Experimental Infection
Source: PLoS Negl Trop Dis. 2012 Aug 14;6(8):e1779. doi: 10.1371/journal.pntd.0001779 (PMC3419193; doi:10.1371/journal.pntd.0001779)

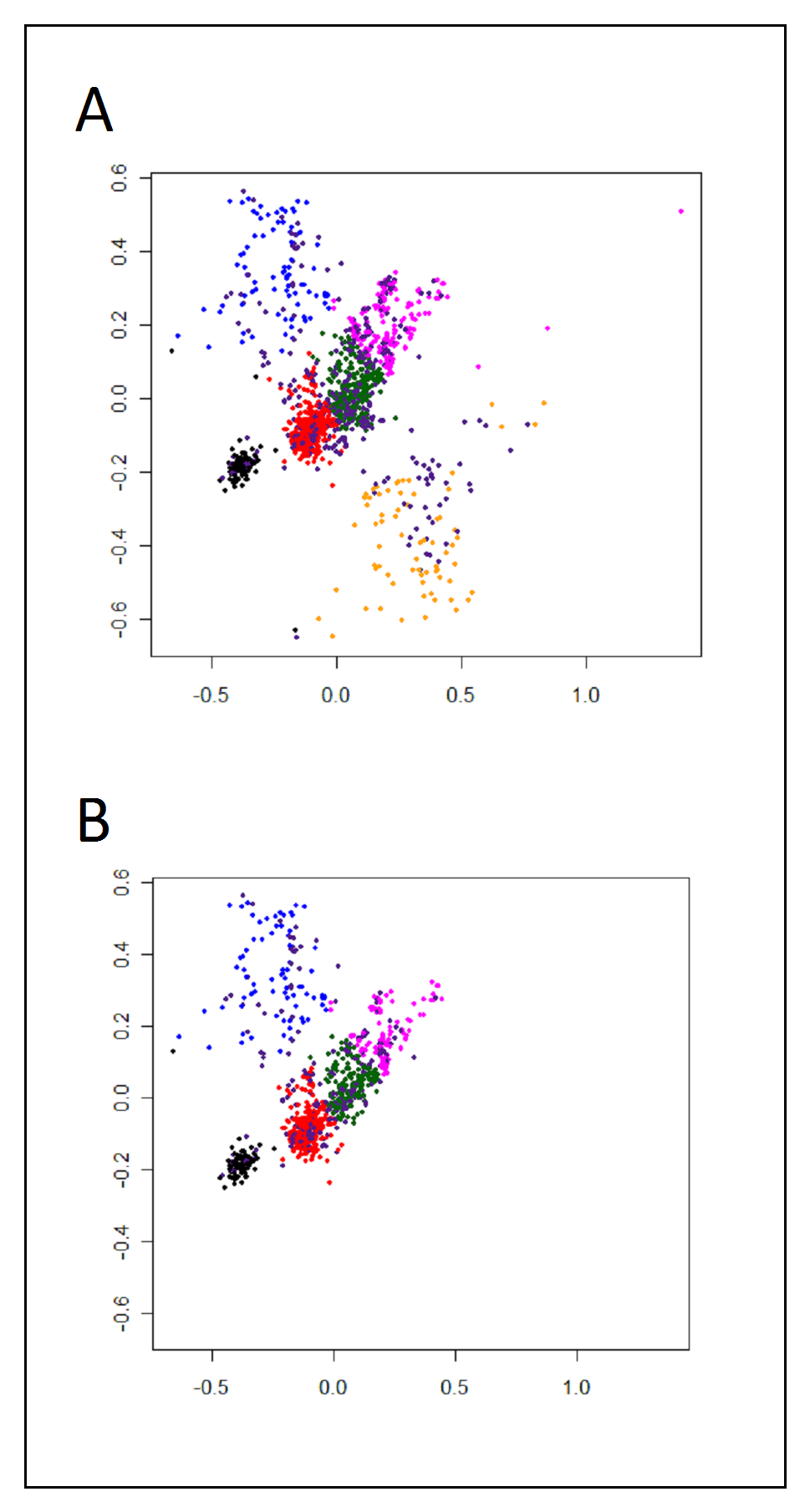

Supplement: Figure S1 — Multidimensional scaling (MDS) plot of MASP genes. Pairwise alignments of the 1,377 MASP genes were performed and the distance matrix was used to generate a multidimensional scaling (MDS) plot. K-means method was used to define the clusters or groups. A: MDS distribution of all 1,377 MASP genes; B: MDS distribution of the 947 MASP genes amplified by e-PCR allowing 2 gaps and 2 mismatches in the primer annealing sequences. Pseudogenes are shown in purple color. (TIF) [file pntd.0001779.s001.tif]

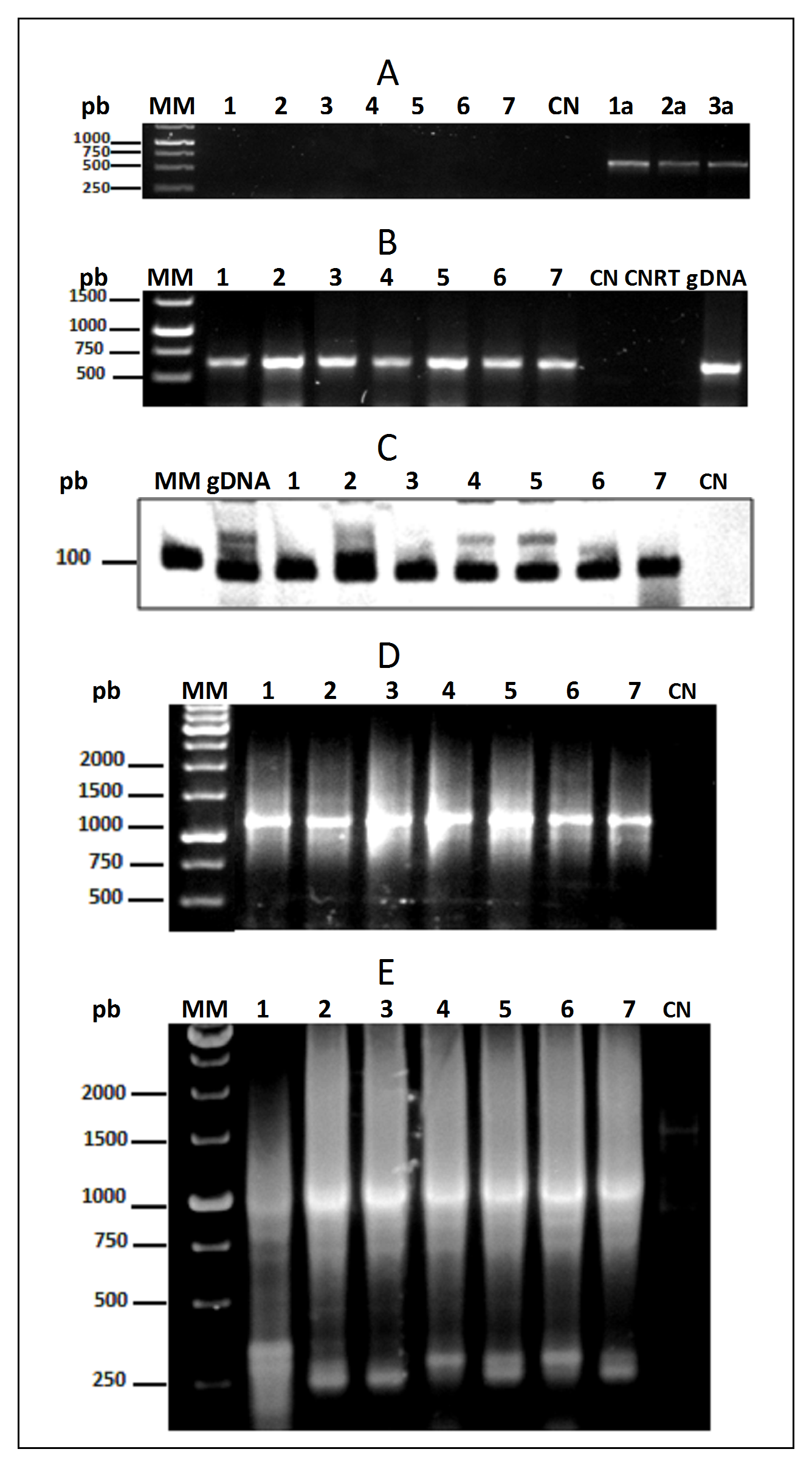

Supplement: Figure S2 — Construction of the MASP expression libraries: RNA and cDNA quality controls and semi-nested RT-PCR for MASP representative agarose (A, B, D, and E) or polyacrilamide (C), electrophorese gels. A. 1 to 7: PCR for AHADH2 gene using total RNA as template; 1a, 2a, and 3a: same RNA samples before DNAse I digestion, DNA contaminated. B. 1 to 7: PCR for AHADH2 gene using the cDNAs as templates corresponding to each RNA sample; CNRT: PCR using a negative cDNA (with no RNA) as template; C. 1 to 7: PCR for RAD51 gene using the cDNAs as templates corresponding to each RNA sample. D. 1 to 7: First PCR reaction for MASP family using each cDNA as template, with primers SL and 3′UTR1. E. 1 to 7: Second PCR reaction for MASP family using each PCR sample as template, with primers SL and 3′UTR2; gDNA: PCR using 10 ng T. cruzi genomic DNA as template; CN: negative control of PCR reaction, with no DNA template. (TIF) [file pntd.0001779.s002.tif]

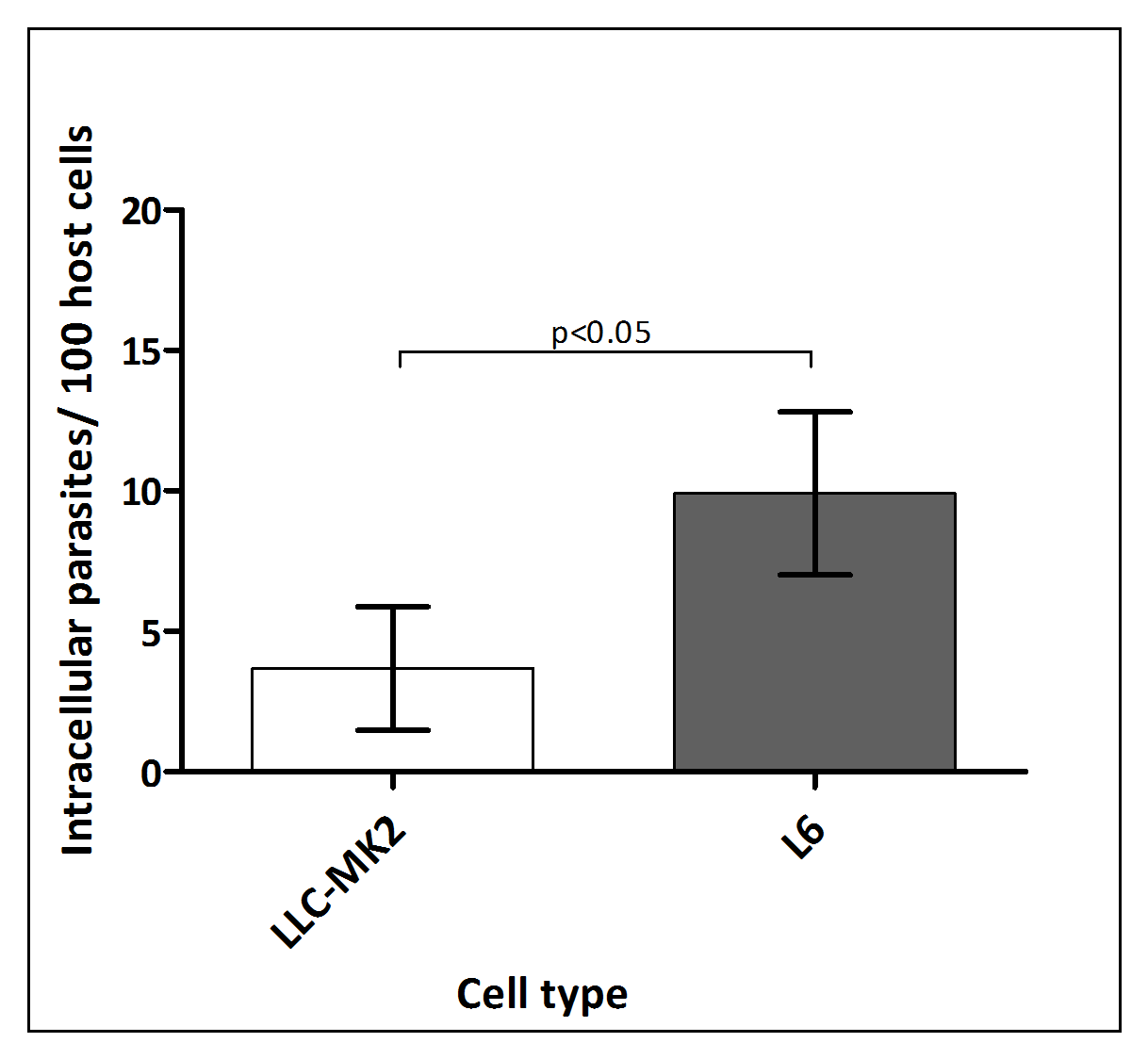

Supplement: Figure S3 — Invasion assay. LLC-MK2 or L6 cells were infected with trypomastigotes derived from L6 cells after 17 passages, fixed, and processed for immunofluorescent detection of intracellular parasites. The data correspond to the mean of triplicates ± SD and were analyzed using the Student's t test. The results are representative of one of two experiments that yielded similar results. (TIF) [file pntd.0001779.s003.tif]

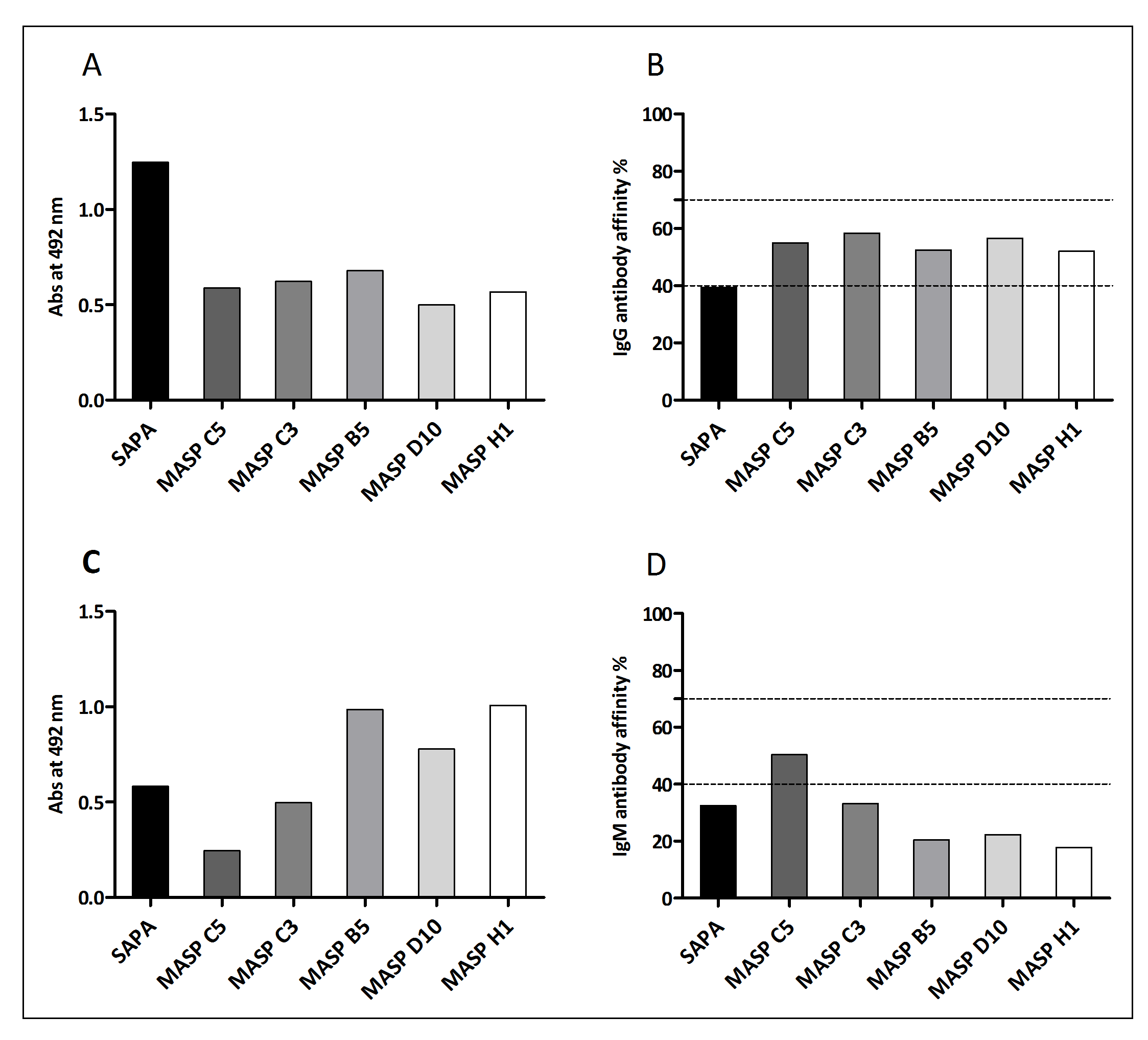

Supplement: Figure S4 — Affinity ELISA of MASP peptides. After the incubation with sera pool of mice infected with T. cruzi after two passages, a single wash step was added to the peptide ELISA protocol with 6 M urea. The results of absorbance were compared to the respective non-washed samples (A and C) in the same experiment. Affinity levels <40% and >40% were considered intermediate and low affinity indexes, respectively (dotted lines). A and B: total reactivity (A) and affinity levels (B) of IgG antibodies against the MASP peptides; C and D: total reactivity (C) and affinity levels (D) of IgM antibodies against the MASP peptides. (TIF) [file pntd.0001779.s004.tif]
