# Supplementary material for: The MASP Family of Trypanosoma cruzi: Changes in Gene Expression and Antigenic Profile during the Acute Phase of Experimental Infection
Source: PLoS Negl Trop Dis. 2012 Aug 14;6(8):e1779. doi: 10.1371/journal.pntd.0001779 (PMC3419193; doi:10.1371/journal.pntd.0001779)
Supplement: Table S1 — List of primers used in the Real Time RT-PCR analysis. (DOC) [file pntd.0001779.s005.doc]

**Table S1:** List of primers used in the Real Time RT-PCR analysis.

| **MASP gene** | **Primer code** | **Primer sequence 5’ – 3’** |
| --- | --- | --- |
| MASP2 | MASP2F (forward) | TGGCGATGATGATGGC |
|  | MASP2R (reverse) | AACCACAGCCGACAC |
| MASP4 | MASP4F (forward) | GGCAAGTGAAACGATTGA |
|  | MASP4R (reverse) | TTCTCTTCTTCTTCCTCCTT |
| MASP14 | MASP14F (forward) | TGATGAAGATGAAGAGATGGA |
|  | MASP14R (reverse) | CTTCCCTGGAACCTGGGT |
| MASP16 | MASP16F (forward) | AGGAAGGCTCATTGAACA |
|  | MASP16R (reverse) | CCTGCTCGGTGTCTG |
| MASP23 | MASP23F (forward) | AGAAGAGGAGACAAAGAAAGA |
|  | MASP23R (reverse) | GCCGCACCAGATGAT |
| MASP27 | MASP27F (forward) | TGGTTCGGGTCAATCG |
|  | MASP27R (reverse) | GGTGACTGTTGCTGTTGG |
| MSH2 | MSH2F (forward) | CGAGCGATTGAATATCTTGT |
|  | MSH2R (reverse) | AGGTGGATGAAATTGTATGC |
| RAD51 | RAD51F (forward) | GGCTGTCAAGGGTATCA |
|  | RAD51R (reverse) | AACCACTGCGGATGTAA |
