# Supplementary material for: The MASP Family of Trypanosoma cruzi: Changes in Gene Expression and Antigenic Profile during the Acute Phase of Experimental Infection
Source: PLoS Negl Trop Dis. 2012 Aug 14;6(8):e1779. doi: 10.1371/journal.pntd.0001779 (PMC3419193; doi:10.1371/journal.pntd.0001779)
Supplement: Table S3 — List of soluble peptides used in ELISA experiments. (DOC) [file pntd.0001779.s007.doc]

**Table S3:** List of soluble peptides used in ELISA experiments.

| **Gene** | **Peptide code** | **Peptide sequence** | **Purity (%)** |
| --- | --- | --- | --- |
| MASP (Tc00.1047053508759.60) | C5 | RAEAPQAPSDTPPGN | 97% |
| MASP4 (Tc00.1047053508541.110) | C3 | ETTPAASPGNTSDGN | 98% |
| MASP27 (Tc00.1047053506615.100) | B5 | GRQAHGSEESGSGQS | 90% |
| MASP23 (Tc00.1047053511089.19) | D10 | KEEDDADATEVTSAG | 92% |
| MASP (Tc00.1047053508221.420) | H1 | STRPTNGSREGDTDT | 98% |
| MASP (Tc00.1047053508305.50) | H5 | GGRAETPSSPSLETQ | 93% |
| MASP (Tc00.1047053510475.100) | J10 | QEGRQTPQSQVNVPQ | 93% |
| Trans-sialidase repetitive region (LTR) [13] | SAPA | STPVDSSAHGTPSTPC | 96% |
